# The Osteoclast Traces the Route to Bone Tumors and Metastases

**DOI:** 10.3389/fcell.2022.886305

**Published:** 2022-05-13

**Authors:** Sharon Russo, Federica Scotto di Carlo, Fernando Gianfrancesco

**Affiliations:** Institute of Genetics and Biophysics “Adriano Buzzati-Traverso”, National Research Council of Italy, Naples, Italy

**Keywords:** osteoclast bone resorption, skeletal tumors, bone metastases, vicious cycle, tumor dormancy

## Abstract

Osteoclasts are highly specialized cells of the bone, with a unique apparatus responsible for resorption in the process of bone remodeling. They are derived from differentiation and fusion of hematopoietic precursors, committed to form mature osteoclasts in response to finely regulated stimuli produced by bone marrow–derived cells belonging to the stromal lineage. Despite a highly specific function confined to bone degradation, emerging evidence supports their relevant implication in bone tumors and metastases. In this review, we summarize the physiological role of osteoclasts and then focus our attention on their involvement in skeletal tumors, both primary and metastatic. We highlight how osteoclast-mediated bone erosion confers increased aggressiveness to primary tumors, even those with benign features. We also outline how breast and pancreas cancer cells promote osteoclastogenesis to fuel their metastatic process to the bone. Furthermore, we emphasize the role of osteoclasts in reactivating dormant cancer cells within the bone marrow niches for manifestation of overt metastases, even decades after homing of latent disseminated cells. Finally, we point out the importance of counteracting tumor progression and dissemination through pharmacological treatments based on a better understanding of molecular mechanisms underlying osteoclast lytic activity and their recruitment from cancer cells.

## Introduction

Bone is a dynamic tissue that constantly requires removal of old and damaged bone and generation of newly synthesized bone to restore structural integrity. For this purpose, two main cell types have evolved: osteoclasts and osteoblasts. The former are polykaryon of hematopoietic origin, which form as a result of the fusion of mononuclear precursors driven by the production of two pivotal cytokines derived from the marrow microenvironment: the macrophage colony-stimulating factor (M-CSF) and the receptor activator of nuclear factor kappa B ligand (RANKL) (Suda et al., 1992; Teitelbaum, 2000). Osteoclasts possess an efficient machinery responsible for mineral dissolution and degradation of large quantity of organic bone matrix and mineralized cartilage (Boyle et al., 2003). Multiple pathologies are associated with osteoclast dysfunction, including Paget’s disease of bone, where genetic determinants lead to higher sensitivity of osteoclast precursors to pro-differentiation cytokines, formation of giant and hyper-nucleated osteoclasts, and increased ability to resorb bone matrix (Divisato et al., 2016; Scotto di Carlo et al., 2020a). Given the variety of cells present within bone marrow, it is not surprising that the bone is also the site of several tumors and metastases (Coleman et al., 2020). Although it is widely accepted that osteoclasts do not undergo neoplastic transformation, increasing evidence has demonstrated their indirect involvement in the process of tumorigenesis and their support to cancer cells. In this review, starting from the description of physiological role of osteoclasts, we will deepen their involvement in bone tumors and metastases. In particular, we will focus on the effects of these cells on the creation and maintenance of cancer microenvironment and their cooperation with tumor cells.

## Biology of Osteoclasts

**FIGURE 1:**
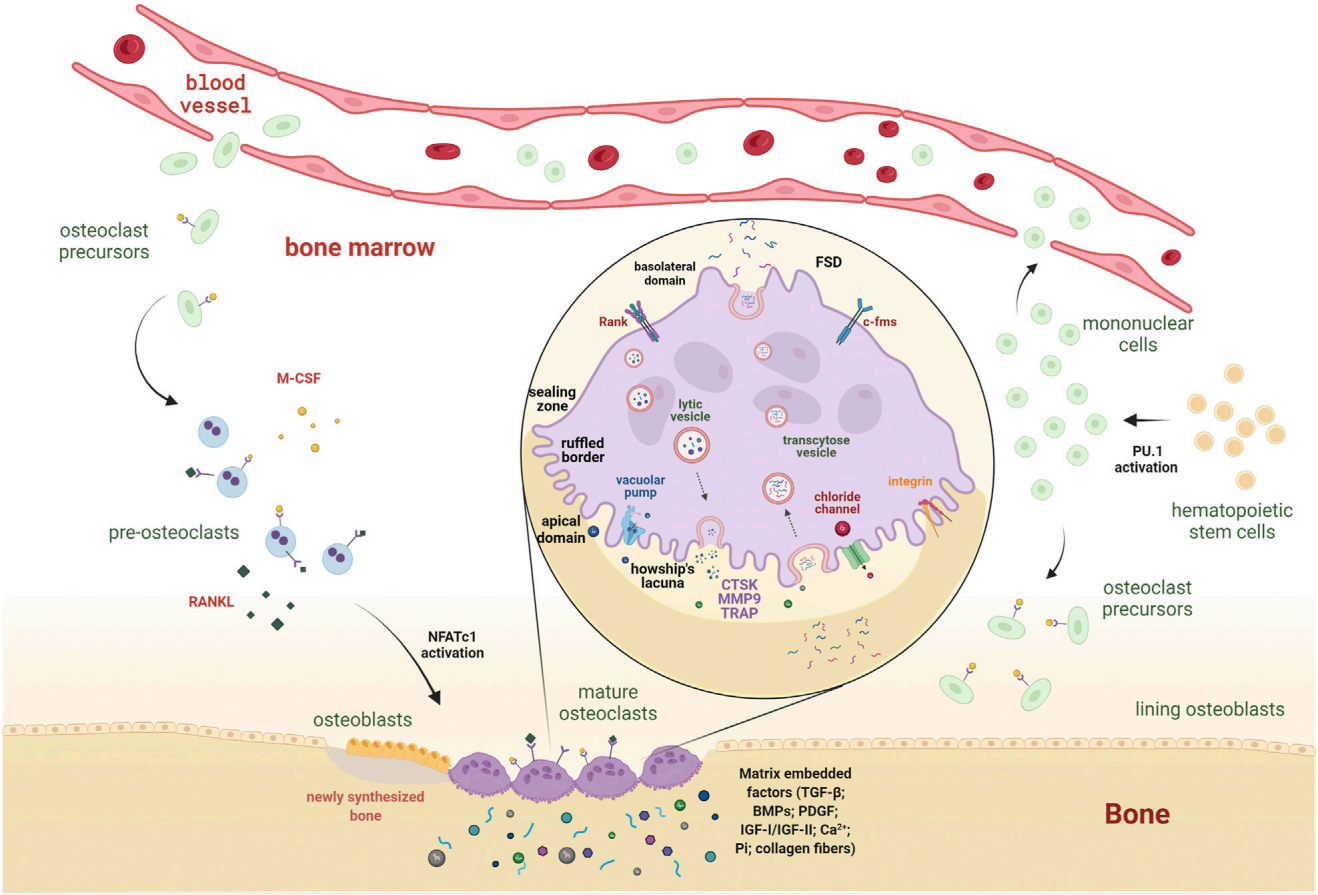
From hematopoietic stem cells to mature osteoclasts: behind the scene of osteoclastogenesis. Following the expression of *PU.1*, hematopoietic stem cells differentiate in mononuclear cells. The latter become osteoclast precursors upon the M-CSF stimulus. They reach the bone site to be resorbed either by recruitment from the surrounding bone marrow or the bloodstream and become mature osteoclasts through stimulation of M-CSF and RANKL, released by the stromal/osteoblastic compartment. The binding of these cytokines to their receptors, c-Fms and RANK, triggers the downstream activation of osteoclastogenic transcriptional factors (e.g., NFATc1), inducing the expression of genes involved in osteoclast-mediated bone erosion. Magnification of mature osteoclast shows functional membrane domains defining an apicobasal polarity. Osteoclasts closely adhere to the bone through the F-actin reach sealing zone, surrounded by integrins (α_v_β_3_), which delineates the ruffled border. The resorption area under the ruffled border, the Howship’s lacunae, is an acidic compartment due to release of H^+^ and Cl^−^ ions. The acidic environment degrades inorganic structures, while organic components require extracellular release of lysosomal enzymes, such as CTSK, MMP9, and TRAP, for their degradation. Osteoclasts release growth and osteotropic factors (i.e., TGF-β, BMPs, PDGF, IGF-I, and IGF-II) trapped within the matrix. Inorganic products (i.e., Ca^2+^ and Pi) and organic molecules (e.g., collagen fibers) are packaged into transcytoses vesicles, which are transported to the functional secretory domain (FSD) at the basolateral domain of osteoclasts and secreted *via* exocytosis. M-CSF, macrophage colony-stimulating factor; RANKL, receptor activator of nuclear factor kappa B ligand; c-Fms, colony-stimulating factor 1 receptor; RANK, receptor activator of nuclear factor kappa B; NFATc1, nuclear factor of activated T cells 1; H^+^, proton; Cl^−^, chloride ion; CTSK, cathepsin K; MMP9, matrix metallopeptidase 9; TRAP, tartrate-resistant acid phosphatase; TGF-β, transforming growth factor-β; BMPs, bone morphogenetic proteins; PDGF, platelet-derived growth factor; IGF-I and IGF-II, insulin-like growth factor I and II; Ca^2+^, calcium ion; Pi, inorganic phosphate.

**Osteoclast formation**. Osteoclastogenesis begins with the stimulation of bone marrow–derived hematopoietic stem cells to turn into mononuclear cells (Tondravi et al., 1997). This phase requires activation of the PU.1 transcription factor, belonging to the Ets family. In fact, mice harboring the *PU.1* gene disruption manifest complete absence of osteoclasts, resulting in osteopetrotic features (Tondravi et al., 1997). Mononuclear cells are either present in the bone marrow and stimulated to become osteoclast precursors or introduced in the bloodstream to circulate until they return to the bone to be resorbed and there differentiate into mature osteoclasts (Muto et al., 2011; Warde, 2011) (Figure 1). The survival, proliferation, and differentiation of osteoclasts and their precursors are provided by M-CSF signaling, which results in the activation of ERK and PI3K/Akt pathways (Nakamura et al., 2001; Glantschnig et al., 2003). Indispensable for osteoclast maturation and their complete activation is RANKL, a type II transmembrane protein produced by osteoblasts as a proteolytically released soluble molecule (Boyle et al., 2003). In response to binding to its receptor RANK, a cascade signaling pathway is triggered, which determines the recruitment of TRAF6 to the intracellular domain of RANK and culminates in the downstream activation of osteoclastogenic transcription factors, such as NF-κB, activator protein 1 (AP-1), cyclic adenosine monophosphate response element–binding protein (CREB), and nuclear factor of activated T cells 1 (NFATc1) (Kobayashi et al., 2001; Gohda et al., 2005) (Figure 1). The process of fusion is essential for the formation of large and multinucleated osteoclasts (Teitelbaum, 2000). Several factors regulate osteoclast fusion, which can be divided into molecules regulated by RANKL (CD9, ATP6V0d2, and DC-STAMP) and those that are not dependent on RANKL stimulation (CD44, CD47, and TREM2) (Xing et al., 2012). Among them, DC-STAMP is the master fusion factor, and its abrogation results in mononuclear TRAP-positive cells, indicating that cell fusion is hampered (Yagi et al., 2005). Mature osteoclasts reach a huge diameter (20–100 µm), essential for the attachment to the site of bone resorption and matrix degradation, drilling a pit into the bone tissue (Tiedemann et al., 2017).

**FIGURE 2:**
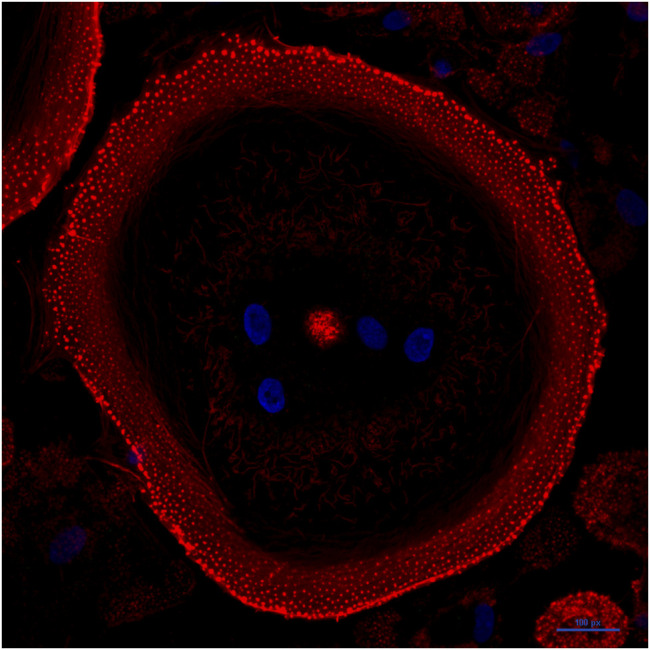
Mature osteoclasts assemble an F-actin ring. Confocal image of a human osteoclast derived from stimulation of peripheral blood mononuclear cells of a healthy donor with M-CSF and RANKL. Nuclei are shown in blue (stained with Hoechst 33342); F-actin is shown in red (stained with phalloidin). The cell is grown on glass and displays a mature actin ring that surrounds the ruffled border and has evolved into a large peripheral podosome belt.

**Osteoclast structure**. Active bone-resorbing osteoclasts show a peculiar cellular polarization, with the apical membrane facing the bone surface—consisting of the *sealing zone* and the *ruffled border*—and the basolateral plasma membrane (Figure 1). The sealing zone is the result of cytoskeleton reorganization aimed at forming an F actin–rich ring to mediate the tight attachment of the osteoclast to the extracellular matrix (Teitelbaum, 2000) (Figure 2). As osteoclast prepares itself to resorb the bone, it forms a ruffled border, a resorbing organelle consisting of intracellular acidified fused vesicles, which represents the site where bone resorption takes place (Blair et al., 1989; Huizing et al., 2008) (Figure 1). Unlike other lytic cells (e.g., macrophages), osteoclasts arrange an extracellular lysosomal acidified compartment, generally called Howship’s lacuna, in which lytic enzymes, such as cathepsin K (CTSK), matrix metalloproteinase-9 (MMP9), and tartrate-resistant acid phosphatase (TRAP), are secreted through protease-bearing vesicles (Abu-Amer et al., 1999; Teitelbaum, 2000; Luzio et al., 2014). Thus, osteoclasts have evolved to utilize lysosomes to carry out one of the most difficult jobs in our body, namely, to excavate the mineralized bone.

**Osteoclast function**. The dissolution of minerals initiates with the secretion of chloride ions (Cl^−^) and the activation of H^+^- and ATP-consuming vacuolar pumps through the ruffled border into the lacuna (Blair et al., 1989) (Figure 1). The chloride ions passively follow protons (H^+^) through the chloride channel present on the ruffled border, and the combined exit of H^+^ and Cl^−^ acidifies the resorption compartment, reaching a low pH environment (∼4) (Blair and Schlesinger, 1990) (Figure 1). The creation of an acidic compartment not only fosters the beginning of matrix dissolution and activation of lytic enzymes (e.g., CTSK) but also directly stimulates osteoclasts, inducing a significant increase in intracellular Ca^2+^ concentration, which acts through calmodulin to stimulate calcineurin activity. Calcineurin, in fact, moves the autoinhibitory domain away from its catalytic site for the dephosphorylation of NFATc1 nuclear localization signal (Park et al., 1995; Wesselborg et al., 1996). As a consequence, the translocation of NFATc1 to the nucleus further stimulates resorption, allowing the expression of osteoclastogenic markers, such as *DC-STAMP, TRAP, CTSK*, and several integrins, which determine full osteoclast maturation (Kobayashi et al., 2001; Mizukami et al., 2002; Komarova et al., 2005). The main enzyme involved in matrix digestion is CTSK, with the participation of other proteolytic enzymes belonging to big families of cysteine proteinases and matrix metalloproteinases (MMPs) (Bossard et al., 1996; Everts et al., 2006). The depletion or inhibition of CTSK results in an overall reduction in bone resorption efficiency interrupting intracellular vesicular trafficking while maintaining all the other osteoclast functions, such as, cell formation, activation, and secretory process (Leung et al., 2011). Of interest, bone site-specific osteoclasts exist, which use a different enzyme repertoire during the bone resorption function (Everts et al., 2009). For example, osteoclasts residing in calvaria or scapular bone, despite expressing cathepsins, preferentially use MMPs for degradation (Shorey et al., 2004; Everts et al., 2006). Another remarkable difference between osteoclast groups that populate different bone sites is the amount of TRAP enzyme released at bone remodeling sites (Everts et al., 2009). Calvarial osteoclasts show increased levels of TRAP activity—up to 25-fold higher—compared to the levels detected at long bone sites, likely to compensate for the aforementioned lower cysteine proteinase activity and also to degrade non-collagenous proteins of this type of bone (Perez-Amodio et al., 2006).

**FIGURE 3:**
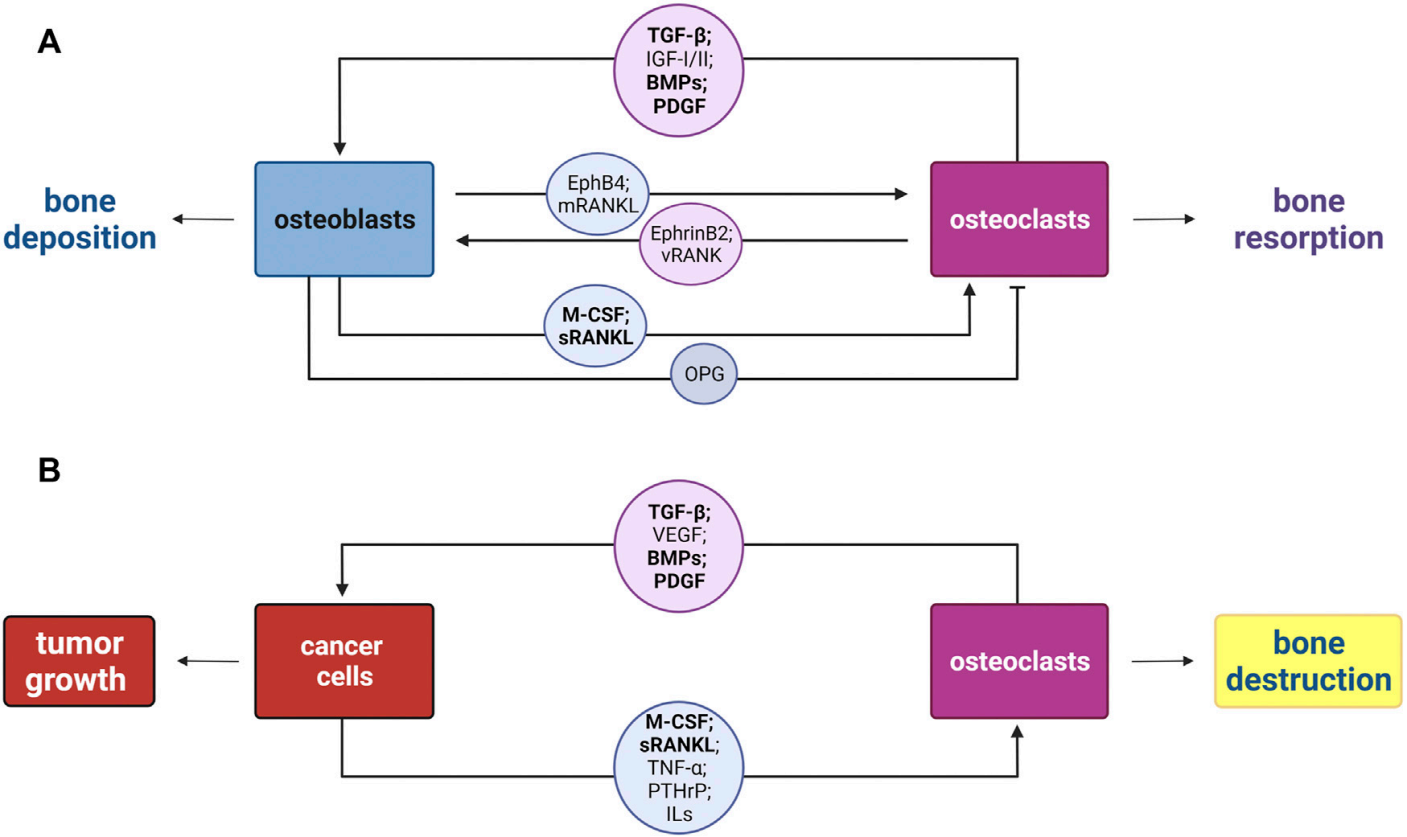
Schematic representation of the osteoclast–osteoblast coupling in bone remodeling *versus* the vicious cycle in bone tumors. **(A)** New bone deposition occurs at bone resorption sites in each cycle of bone remodeling by the osteoblast–osteoclast coupling. Osteoblasts mediate both osteoclasts activation and inhibition, *via* secretion of M-CSF, soluble RANKL (sRANKL), and OPG. Instead, osteoclasts are able to mediate bone deposition by the release of matrix-embedded factors upon bone erosion (TGF-β, IGF-I/II, BMPs, and PDGF), through the secretion of extracellular vesicles carrying the receptor RANK (vRANK), and direct interaction through the membrane protein ephrinB2 with the osteblastic EphB4. **(B)** Disruption of the osteoblast–osteoclast coupling by the vicious cycle of bone. Tumor cells secrete soluble factors including M-CSF, sRANKL, TNF-α, PTHrP, and several interleukins (ILs), which act on osteoclast to enhance their bone destruction activity. Consequently, osteoclasts contribute to tumor growth and progression releasing TGF-β, pro-angiogenic factor VEGF, different BMPs, and PDGF. In bold are highlighted the molecules shared by the two cycles. M-CSF, macrophage colony-stimulating factor; RANKL, receptor activator of nuclear factor kappa B ligand; OPG, osteoprotegerin; TGF-β, transforming growth factor-β; IGF-I and IGF-II, insulin-like growth factor I and II; BMPs, bone morphogenetic proteins; PDGF, platelet-derived growth factor; RANK, receptor activator of nuclear factor kappa B; EphB4 and Eph receptor B4; TNF-α, tumor necrosis factor-α; PTHrP, parathyroid hormone-related protein; VEGF, vascular endothelial growth factor.

**Osteoclast–osteoblast coupling activities**. The osteoclast resorbing activity needs to be neutralized to avoid excessive bone erosion. To this end, osteoblasts—in addition to the aforementioned RANKL—produce Osteoprotegerin (OPG), the soluble decoy receptor for RANKL that selectively inhibits RANK and RANKL binding, due to the homology to the members of the TNF receptor family, thereby blocking osteoclastogenesis (Simonet et al., 1997; Lacey et al., 1998; Robling et al., 2006) (Figure 3A). Thus, osteoblasts can be thought of as regulators of both osteoclast activation and inhibition.

Osteoclasts act upon osteoblast lineage cells producing multiple proteins, either associated with exosomes (e.g., RANK), released from the resorbed bone, or secreted, and they also directly interact with osteoblasts through membrane-bound proteins (Sims and Martin, 2020) (Figure 3A). The ability of osteoclasts to regulate osteoblasts, even independent of their resorption activity, was observed with the formation of mineralized nodules in osteoblast cultures in the presence of conditioned medium derived from osteoclasts grown on plastic, and hence not releasing products from degradation (Karsdal et al., 2008). Nevertheless, the byproducts of bone degradation certainly impact osteoblast differentiation. Indeed, released transforming growth factor-β (TGF-β) is able to promote the recruitment of BMSCs at the remodeled site, by activating mammalian target of rapamycin (mTOR) (Tang et al., 2009). Also, the release of insulin-like growth factors, that is, IGF-I and IGF-II, together with BMPs and the homodimeric platelet-derived growth factor (PDGF) additionally support BMSCs recruitment and stimulate osteoblast progenitor expansion, migration, and differentiation, thus enhancing bone formation (Mitlak et al., 1996; Xian et al., 2012; Xie et al., 2014). Furthermore, a recent seminal work highlighted that the RANK and RANKL axis works as *reverse signaling* to allow osteoclasts to communicate with osteoblasts, utilizing the same molecules, which they are target thereof (Ikebuchi et al., 2018). Osteoclasts release small extracellular vesicles that carry the receptor RANK (vesicular RANK), which binds RANKL molecules present on pre-osteoblast and early osteoblast membranes (membranous RANKL) (Ikebuchi et al., 2018). This binding triggers intracellular signaling, culminating in the expression of the master regulator of osteoblastogenesis, *RUNX2* (Ikebuchi et al., 2018; Zaidi and Cardozo, 2018). Of note, the proline-rich motif in the RANKL cytoplasmic tail is fundamental for the reverse signaling to occur. In fact, mice harboring a point mutation in this domain showed reduced activation of osteoblasts stimulated by RANK-exposed microvesicles released by osteoclasts (Ikebuchi et al., 2018). Despite the ability to regulate osteoblasts at a distance, osteoclasts also developed mechanisms to directly interact with osteoblast precursors. An example of this class of direct cell–cell communication is EphrinB2–EphB4, where osteoclast-derived ephrinB2 contact with osteoblastic EphB4 enhances osteoblast differentiation, by lowering RhoA activity (Zhao et al., 2006).

Once completed bone deposition, osteoblasts remain trapped within the matrix and become osteocytes, showing extensive dendritic processes through which they communicate with other bone cells. Indeed, osteocytes are the main cells able to sense mechanical and hormonal stimuli, to which they respond by regulating osteoblast and osteoclast activity (Bellido, 2014). In this regard, osteocytes express the RANKL cytokine to support osteoclastogenesis, even in a higher amount than bone marrow stromal cells (Nakashima et al., 2011). Accordingly, mice lacking RANKL specifically in osteocytes manifest a dramatic osteopetrotic phenotype (Nakashima et al., 2011).

Therefore, osteoclast and osteoblast formation and activity are strictly regulated to ensure a physiological bone remodeling and the maintenance of the quality and quantity of bone through the entire life-course, where osteoclastic bone erosion is generally always followed by osteoblastic bone deposition (Martin, 2014).

## Role of Osteoclasts in Tumor Biology

### Primary Bone Tumors

Although most bone cancers have a mesenchymal origin, osteoclasts frequently contribute to increase their aggressiveness, conferring lytic features to tumors (Valastyan and Weinberg, 2011). The pivotal role of osteoclasts in the creation and maintenance of a favorable tumor microenvironment stems from initial studies in animal models supporting this evidence. Mice harboring a deletion in the integrin β3—an essential protein for osteoclast activity—manifest resistance to bone tumor growth and osteolytic metastasis upon intra-tibial injection of cancer cell lines, which conversely promptly caused tumor-associated bone loss and tumor metastasis in the control group (Bakewell et al., 2003).

Among the bone tumors with a remarkable osteoclast activity, giant cell tumors (GCT), osteosarcomas (OS), and chondrosarcomas (CS) are the most common. These tumors have different origin and behavior, yet they share a low-to-high composition of osteoclasts that mediate the tumor-associated bone destruction.

**Giant cell tumor**. Giant cell tumor is a common benign tumor, mainly affecting the epiphysis of long bones in young adults (Goldenberg et al., 1970). Although the neoplastic cell has been undoubtedly identified as the stromal cell carrying mutations in *H3F3A*, osteoclast-like giant cells strongly influences the aggressiveness of the tumor, to the extent that these cells name the tumor (Behjati et al., 2013). These giant osteoclastic cells could comprise more than 50% of the total cell content of the tumor (Atkins et al., 2000; Morgan et al., 2005). Giant cells, albeit non-tumoral elements, are responsible for destructive osteolysis seen in GCT, even involving the bone cortex and resulting in pathological fractures in ∼30% of patients (Campanacci et al., 1987). The lytic properties of osteoclast-like giant cells also allow the tumor to extend into soft tissues (Mavrogenis et al., 2017; Palmerini et al., 2019). Similar to osteoclasts, giant cells also express the RANK receptor on their surface, suggesting their formation as a result of fusion of monocytic precursors recruited by the stromal cells through the expression of RANKL, exactly as is the case for osteoclasts (Morgan et al., 2005). The high expression of RANKL within the tumor paved the way for the use of denosumab, a fully human monoclonal antibody to RANKL, as efficient treatment for GCT, reducing its lytic activity (Chawla et al., 2019; Alothman et al., 2020). Therefore, targeting giant cells is a more effective approach than targeting neoplastic stromal cells. GCT can also arise as severe degeneration of Paget’s disease of bone (PDB), a bone remodeling disorder (GCT/PDB). GCT/PDB generally involves the axial skeleton and is caused by germline mutations in *ZNF687*, thus affecting both the stromal and osteoclastic compartments (Divisato et al., 2016; Divisato et al., 2018). *ZNF687* encodes a transcription factor with a key role in promoting osteoclast differentiation (Divisato et al., 2018). Consequently, giant cells found in GCT/PDB biopsies are even bigger than conventional GCT, and the tumor usually shows a worse prognosis (Divisato et al., 2017; Scotto di Carlo et al., 2020b). PDB patients harboring *ZNF687* mutations display a lower likelihood of developing the tumor when treated with bisphosphonates, strong inhibitors of osteoclast activity used to suppress the elevated bone resorption in PDB (Rendina et al., 2015; Divisato et al., 2018; Ralston, 2020). It seems that by controlling the progression of PDB, the neoplastic transformation is prevented (Scotto di Carlo et al., 2020b). This observation confirms that tumor development and aggressiveness might be reduced by decreasing osteoclast resorption.

**Osteosarcoma**. Osteosarcoma (OS) is the most common and aggressive bone tumor, primarily arising in long bones of children and adolescents (Mirabello et al., 2009). The great genetic heterogeneity that characterizes OS results in multiple histological appearances of the tumor (Kansara et al., 2014; Rickel et al., 2017). Common histological variants of OS include osteoblastic, chondroblastic, and fibroblastic types, depending on the main cellular atypia and the type of the extracellular matrix produced by neoplastic cells (Kansara et al., 2014). Giant cell OS is a rare histological variant (comprising less than 3% of all OS), characterized by numerous osteoclast-like giant cells in addition to osteoid matrix. In addition to making differential diagnosis difficult due to similarity with other giant cell rich lesions (e.g., GCT and aneurysmal bone cyst), the presence of osteoclasts within OS is a key driver of cancer-associated bone degradation (Domansk and Walther, 2017). However, bone degradation is part of the pathological process for all OS subtypes, and a certain number of osteoclasts are consistently present in OS, both at the periphery of the tumor and within the tumor tissue (Avnet et al., 2008). High levels of PTHrP, IL-6, and RANKL have been detected in both OS samples and cell lines, suggesting local production of cytokines and growth factors as a mechanism for tumor cells to enhance osteoclastogenesis (Avnet et al., 2008). In support of this, OS-derived exosomes promote osteoclast differentiation and bone resorption activity (Raimondi et al., 2020). Under this respect, PTHrP seems to be of particular importance: through its secretion, the tumor cells can simultaneously stimulate the expression of RANKL and reduce the expression of OPG by osteoblast cells, overstimulating osteoclast differentiation (Guise et al., 2002). Consequently, it appears evident that targeting RANKL is one of the most suitable approaches in personalized medicine in order to contrast OS aggressiveness (Trinidad and Gonzalez-Suarez, 2016). Mice with osteolytic OS treated with OPG or RANK-Fc, a therapeutic antagonist for RANKL, showed decreased osteoclast number, although the therapy had no effect on cancer cells. However, OS-bearing mice displayed reduced tumor growth, increased survival, and significant reduction in tumor metastasis degeneration. In addition, preventive treatment with RANK-Fc completely inhibited OS development (Lamoureux et al., 2007; Lamoureux et al., 2008; Chen et al., 2015). To further underscore the importance of RANKL signaling in OS, crossing *Rankl* ^*−/−*^ mice with a mouse model with inactive p53 and Rb1, which spontaneously develops aggressive and metastatic OS, resulted in a marked suppression of OS development and reduced number of metastatic lesions (Chen et al., 2015). This emphasizes the role of bone-resorbing osteoclasts in the progression of OS.

In the elderly, OS typically associates with PDB (OS/PDB) and shows a 5-year survival rate almost nil (Deyrup et al., 2007). OS/PDB usually shows enrichment in osteoclasts within the tumor sites, perhaps as a result of recruitment of pre-existing hyperactive osteoclasts in the PDB sites (Hansen et al., 2006). Although genetically complex, the loss of the *PFN1* has been found as a recurrent theme in OS/PDB samples (Scotto di Carlo et al., 2020a; Scotto di Carlo et al., 2022). *PFN1* encodes Profilin 1, an essential actin-binding protein with a key role in cytoskeleton remodeling and intracellular trafficking (Pimm et al., 2020; Murk et al., 2021). Profilin 1 depletion in the RAW264.7 monocytic cell line resulted in higher sensitivity to RANKL and consequent formation of large and hyper-nucleated osteoclasts (Scotto di Carlo et al., 2020a). Accordingly, a mouse model carrying a loss-of-function mutation in *Pfn1* exhibits active bone resorption and bone loss (Wei et al., 2021). This underscores the effect of Profilin 1 downregulation in osteoclast formation in OS.

**Chondrosarcoma and chondroblastoma**. Chondrosarcomas are the second most common malignant bone sarcomas and constitute a heterogenous group of neoplasms where tumor cells produce cartilage matrix (Gelderblom et al., 2008; Valery et al., 2015). The main localization areas of chondrosarcomas are pelvic bone, scapula, and long bones (Brown et al., 2018). The bone microenvironment plays a pivotal role in chondrosarcoma development, as supported by histological examination of conventional chondrosarcoma revealing the presence of numerous bone cells (i.e., osteoclasts and osteoblasts) in close contact to the cartilaginous tumor cells (Grimaud et al., 2002). The infiltration of chondrosarcoma tumor cells into the bone tissue is associated with bone resorption through the stimulation of osteoclast formation (David et al., 2011). Accordingly, the presence of osteoclasts has been observed in the microenvironment of chondrosarcoma, responsible for the bone destruction behavior (David et al., 2011; Otero et al., 2014). Inherent in this observation, chondrosarcoma possesses an aggressive lytic activity, characterized by osteopenia, cortical erosion, and pathologic fractures, as detected by radiographic imaging (Ollivier et al., 2003; Littrell et al., 2004; Gelderblom et al., 2008). Because tumor cells lack the ability to resorb the bone, they take advantage of osteoclast activation in the tumor microenvironment for their propagation. Indeed, culture media conditioned by chondrosarcoma cell lines was able to trigger the formation of mature osteoclasts in the RAW264.7 monocytic cell line. Also, chondrosarcoma tumors formed by the injection of chondrosarcoma cells in nude mice contained a remarkable number of TRAP-positive osteoclasts (Clark et al., 2010). Furthermore, unlike subcutaneously formed chondrosarcomas, the intra-tibial tumors displayed increased tumor infiltration and bone destruction (Hamm et al., 2010). Interestingly, in these tumors high expression of osteoclast-related enzymes was detected, for example, cathepsins and metalloproteineases, presumably promoting tumor invasion (Hamm et al., 2010). In line with an osteoclast-promoting capacity of chondrosarcoma tumor cells, therapies aimed at targeting RANKL proved to be beneficial in chondrosarcoma treatment. Indeed, chondrosarcoma is highly resistant to current chemotherapy and radiation regimens, and surgical treatment leads to severe disability (Moussavi-Harami et al., 2006; Polychronidou et al., 2017). Therefore, the use of bisphosphonates as adjuvant therapy may have clinical utility in chondrosarcoma patients. In particular, two different studies demonstrated that treatment of chondrosarcoma-bearing rats with zoledronic acid—a potent bisphosphonate—prevented cortical destruction, inhibited trabecular resorption, and resulted in decreased tumor volume in the bone (Gouin et al., 2006; Otero et al., 2014). Thus, suppression of osteoclasts seems to be a key approach to inhibit local cancer growth.

Another bone tumor–producing chondroid matrix is chondroblastoma. However, chondroblastoma is a benign tumor, even though it is locally aggressive and affects younger patients (Kaim et al., 2002; McCarthy and Frassica, 2014). It characteristically occurs in the epiphyses of long bones, particularly the femur and tibia (McCarthy and Frassica, 2014). Histologically, chondroblastomas exhibit multinucleated osteoclast-like giant cells (Lucas, 1996). For this reason, it resembles giant cell tumor; in fact, in 1931, it was defined as “chondromatous giant cell tumor” (De Mattos et al., 2013). However, immunohistochemical positivity to protein S-100, marker of chondrocytes and cartilaginous differentiation, exclusively in chondroblastoma tumor cells, helps the differential diagnosis between the two tumors (Monda and Wick, 1985; Nielsen et al., 2017). As expected, the presence of osteoclastic giant cells is responsible for the aggressive osteolytic characteristic of this tumor. In agreement with an osteoclast recruitment and consequent osteolytic bone destruction, RANKL, but not OPG, expression is found at high levels in chondroblastoma specimens, and denosumab has been used neoadjuvantly for the treatment of chondroblastomas with success (Huang et al., 2003; Visgauss et al., 2021).

**FIGURE 4:**
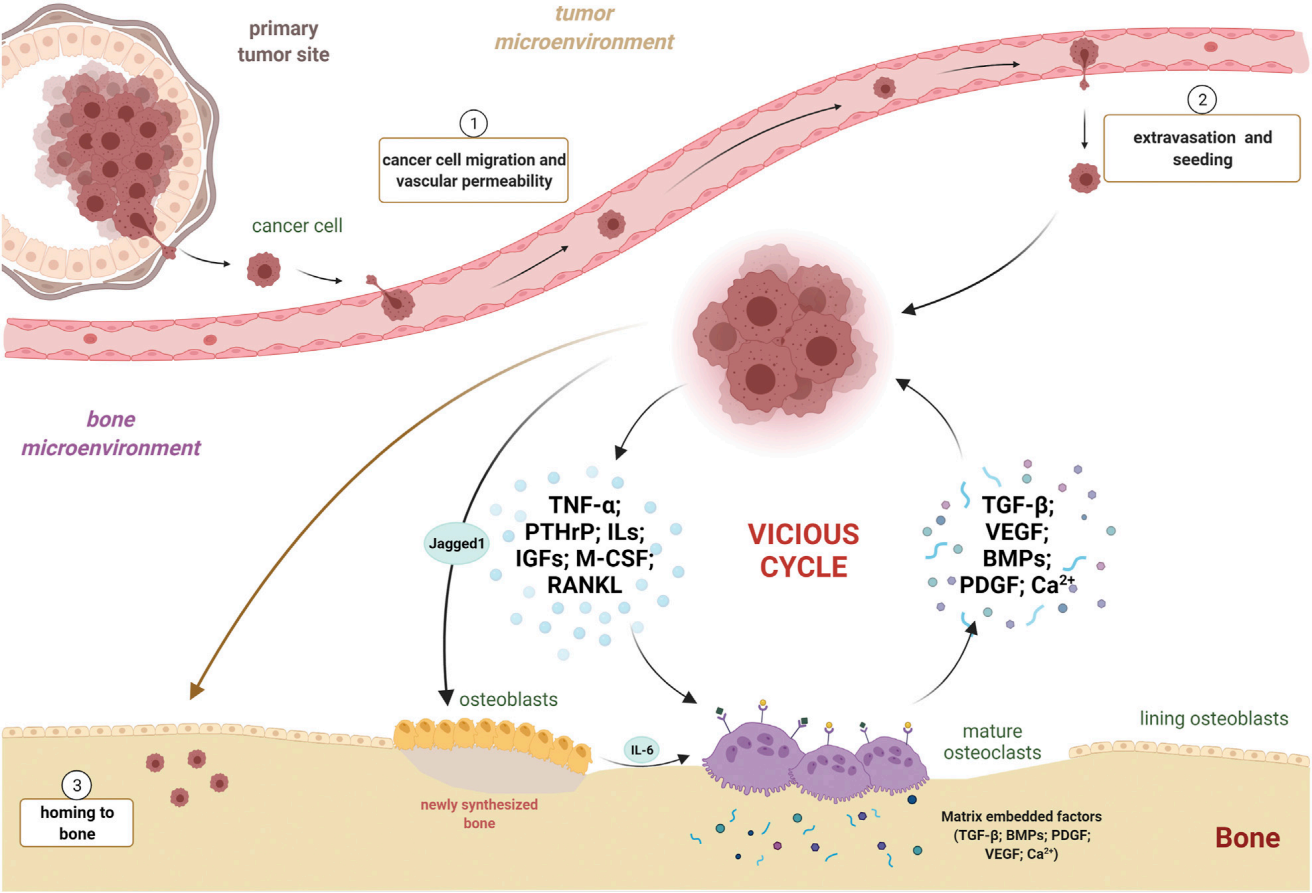
Osteoclasts and cancer cells: partners in crime in the vicious cycle. Three principal steps are necessary for a bone metastasis to occur: 1) escape of the cancer cell from primary tumor site, migration, and vascular permeabilization; 2) extravasation from blood stream and bone marrow seeding; and 3) colonization and homing to bone. Once engrafted in the bone, neoplastic cells secrete pro-osteoclastogenic factors, such as TNF-α, PTHrP, ILs, IGFs, M-CSF, and RANKL, promoting osteoclast differentiation and activity. Jagged1 expression by neoplastic cells fuels IL-6 secretion by osteoblasts, exacerbating osteoclastogenesis and tumor growth. Mature osteoclasts, in turn, free several pro-tumorigenic factors, such as TGF-β, VEGF, BMPs, PDGF, and Ca^2+^ ions, all capable of stimulating tumor growth. This leads to the instauration and fueling of the vicious cycle between cancer cells and active osteoclasts. TNF-α, tumor necrosis factor-α; PTHrP, parathyroid hormone-related protein; ILs, interleukins; IGFs, insulin-like growth factors; M-CSF, macrophage colony-stimulating factor; RANKL, receptor activator of nuclear factor kappa B ligand; TGF-β, transforming growth factor-β; VEGF, vascular endothelial growth factor; BMPs, bone morphogenetic proteins; PDGF, platelet-derived growth factor; Ca^2+^, calcium ion.

**Multiple myeloma**. Multiple myeloma is a hematologic malignancy characterized by the accumulation of malignant plasma cells in the bone marrow due to a tropism for the bone medullary compartment, leading to impaired hematopoiesis (Terpos et al., 2018). Osteolytic bone disease is the hallmark of multiple myeloma that severely undermines the life quality of patients (Edwards et al., 2008). Similar to the process described for other tumor types, a vicious cycle exists when myeloma cells home to the marrow and release cytokines and factors that induce osteoclast activity and bone destruction, including TNF-α, IL-1, IL-3, and IL-6 (Figures 3B, 4) (Edwards et al., 2008; Kawano et al., 1988; Lee et al., 2004). Osteoclasts contribute to multiple myeloma through different mechanisms. In addition to promoting bone destruction and, consequently, tumor growth, osteoclast activity promotes angiogenesis, required for cell survival and proliferation. Indeed, osteoclastic bone resorption releases the vascular endothelial growth factor (VEGF) from the bone matrix through the production of MMP9 (Figure 4) (Raje et al., 2019; Roodman, 2009; Mansour et al., 2017). Accordingly, zoledronate treatment to mice developing multiple myeloma after the intravenous injection of myeloma cells resulted in decreased osteolysis, tumor burden, and angiogenesis (Croucher et al., 2003). Furthermore, osteoclasts are able to remodel the endosteal niche within the bone marrow, thus activating dormant myeloma cells (Lawson et al., 2015). Last, osteoclasts are capable of inducing T cell apoptosis or suppression, maintaining an immune suppressive environment in multiple myeloma, *via* direct inhibition of proliferating CD4^+^ and CD8^+^ T cells. In addition, during osteoclastogenesis several molecules are upregulated, including Galectin-9, which specifically induces apoptosis of T cells while sparing monocytes and myeloma cells (An et al., 2016). Therefore, in multiple myeloma, osteoclasts participate in the regulation of angiogenesis, remodeling of marrow niches, and control of immune response; this creates a vicious cycle in which the bone resorptive process increases tumor burden, perpetuating the cycle (Figure 4). As one would expect, multiple myeloma samples and cell lines express high levels of RANKL, and mouse models of multiple myeloma exhibit deregulated RANKL/OPG balance (Pearse et al., 2001; Roux et al., 2002; Raje et al., 2019). Therefore, therapeutic options for multiple myeloma patients target bone remodeling. In addition to bisphosphonates and denosumab, hitherto described for other osteoclast-rich bone lesions, proteasome inhibitors have also been used in the clinical regimens for multiple myeloma (Morgan et al., 2013; Raje et al., 2016; Gandolfi et al., 2017; Raje et al., 2019). By preventing the degradation of IκBα, they decrease NF-κB expression and inhibit osteoclast differentiation and function (Kupperman et al., 2010). Indeed, the treatment with proteasome inhibitors in multiple myeloma patients decreased serum levels of both RANKL and bone resorption markers (Terpos et al., 2006).

### Skeletal Metastases

Bone marrow is a frequent site of metastasis for a number of cancers—including breast, prostate, and lung cancer—and bone metastases are generally associated with increased morbidity and mortality (Harding et al., 2018; Weilbaecher et al., 2011). In order to metastasize, tumor cells must penetrate the blood or lymphatic circulatory system, where they exhibit a non-proliferative quiescent state and are arrested in G0-G1 (Fares et al., 2020). When tumor cells home to bone marrow, they encounter a unique microenvironment that contains a variety of cell types and growth factors that support their colonization (Wang et al., 2015; Yu-Lee et al., 2018) (Figure 4). Upon dissemination into the bone marrow, tumor cells may either grow as overt metastasis or enter a dormant state. The majority of dormant tumor cells enter a quiescent, non-proliferative state by exhibiting mitotic arrest through reversible G0-G1 arrest. Therefore, they remain viable but do not proliferate (Park and Nam, 2020). However, another way to reach dormancy is through a constant balance between proliferation and apoptosis, where tumor cells divide but do not increase in number. This occurs mainly when a dormant tumor cell grows into a micrometastasis and requires new vasculature: if angiogenesis is suppressed, poor vascularization leads to cell death (“angiogenic dormancy”) (Sosa et al., 2014). Moreover, in the so-called “immune-mediated dormancy,” the immune system keeps the number of proliferative tumor cells unchanged mostly via cytotoxic activity of CD8^+^ cells (Romero et al., 2014).

**Role of osteoclasts in bone metastases**. The bone does not receive invading cancer cells passively. In fact, primary tumor cells selectively and actively prime the host microenvironment to promote the formation of a pre-metastatic niche. Disseminating cancer cells release factors and extracellular vesicles that induce vascular leakage, extracellular matrix remodeling, and immunosuppression (Peinado et al., 2017). For example, tumor cells secrete the parathyroid hormone-related peptide (PTHrP) to promote osteoclast differentiation and activity, by altering osteoblast production of RANKL and its antagonist OPG (Guise et al., 1996). The resultant bone degradation releases a number of growth factors embedded in the bone matrix, such as TGF-β, which further stimulates the malignancy of tumor cells (Buijs et al., 2012). Thus, tumor metastasis to bone is a complex process involving reciprocal interplay between cancer cells and host stroma (Figure 4). Another essential microenvironmental factor is represented by hypoxia, which activates signaling through hypoxia-inducible factor 1 (HIF-1) in response to low oxygen levels. Widely accepted, hypoxia strongly stimulates osteoclasts differentiation and activity *via* the regulation of RANKL/OPG ratio (Bozec et al., 2008). Previous studies have proven that activation of HIF-1 signaling in breast cancer cells fosters bone colonization and osteolysis following intracardiac and orthotopic injections of these cells (Hiraga et al., 2007; Lu et al., 2010). For example, mice injected with human breast cancer cells constitutively expressing active HIF-1 exhibited a more aggressive tumor growth and a greater osteolysis in long bones (Hiraga et al., 2007). It has been demonstrated that hypoxia is specifically related to bone metastasis in patients with estrogen-receptor negative breast cancer, where the analysis of breast cancer cell secretome identified lysil oxidase significantly associated with bone tropism and relapse (Cox et al., 2015). Interestingly, lysil oxidase was shown to promote NFATc1-dependent osteoclastogenesis, independent of RANK ligand, to favor bone resorption and provide disseminating cancer cells with a platform to colonize and form metastases (Cox et al., 2015). The evidence that bone-degrading osteoclasts aid the expansion of breast cancer metastatic lesions also came by the observation that circulating tumor cells express high levels of the Notch ligand, Jagged1 (Reedijk et al., 2005). Remarkably, Jagged1 promotes tumor growth by stimulating IL-6 release from osteoblasts, directly activating osteoclast differentiation and activity (Figure 4) (Sethi et al., 2011). Therefore, bone resorption appears to mediate proliferation of metastatic tumor cells within bone marrow. In fact, OPG treatment in mice decreased bone resorption and, importantly, significantly reduced tumor area and overall cancer-associated sclerotic bone lesion area after intra-tibial implantation of human breast cancer cells (Zheng et al., 2008). Intriguingly, a calcium deficient diet increased the levels of bone resorption and, in parallel, the size of tumors and osteosclerotic areas, confirming the effect of bone resorption in mediated tumor growth (Zheng et al., 2008). Breast cancer is not the only malignant tumor showing a substantial tropism for bone in metastatic process. Nearly 80% of patients with advanced-stage prostate cancer develop skeletal metastases, and this feature is associated with poor prognosis, with less than 50% of patients surviving 1 year after diagnosis of bone metastasis (Halabi et al., 2016; Macedo et al., 2017). Although bone metastases in prostate cancer patients are primarily sclerotic, and therefore characterized by deposition of new bone by osteoblasts, a critical role for osteoclasts has been described in the process of tumor growth (Macedo et al., 2017). Indeed, osteoclast precursors isolated from the bone marrow of C57BL6 mice were able to fully differentiate into mature osteoclasts in the presence of conditioned medium of prostate cancer cell lines, suggesting that tumor cells secrete factors needed to promote osteoclastogenesis (Polavaram et al., 2021). In support of this observation, castration-induced bone loss in nude mice triggered growth of tumor cells within the skeleton after intracardiac injection of prostate cancer cells, even in 12-week-old animals where the low rate of bone turnover generally leads to only a moderate skeletal tumor growth (Ottewell et al., 2014). Similarly, ovariectomy of C57BL6 mice performed 1 week before the inoculation of the multiple myeloma cells in the tail vein simultaneously increased bone remodeling and accelerated the severity of the tumor, with an earlier development of osteolytic lesions in tibiae and femurs (Libouban et al., 2003). Further confirming an active role for osteoclasts in skeletal metastases, mice administered granulocyte colony-stimulating factor (G-CSF) demonstrated increased markers of osteoclast activity, decreased bone mineral density, and also significantly increased tumor growth in the marrow cavity after intra-tibial injection of melanoma cells (Hirbe et al., 2007). Therefore, osteoclasts are predominant actors in bone metastasis formation, mediating bone degradation and promoting skeletal tumor growth.

**Role of osteoclasts in reactivation from tumor dormancy**. Dormant cells exhibit prolonged survival in cell cycle arrest (for up to several decades) and have the potential ability to exit this state and start proliferating again, eventually leading to overt metastatic disease. Until that moment, they are clinically undetectable (Park and Nam, 2020). Furthermore, the lack of proliferation provides dormant cells with an inherent resistance to cytotoxic treatments, for example, chemotherapies and radiation, which generally target dividing cells (Santos-de-Frutos and Djouder, 2021). The notion that the bone might provide dormancy-inducing factors stems from the evidence that in prostate cancer patients, bone metastasis can occur years or decades after prostatectomy, suggesting that disseminated tumor cells had been dormant at the metastatic site in bone (Ahove et al., 2010; Yu-Lee et al., 2018; Yu-Lee et al., 2019). Central to the mechanisms of cellular dormancy and reactivation is the crosstalk between cancer cells and their microenvironment (Fares et al., 2020).

A key dormancy-promoting role is thought to be played by osteoblasts, which support cancer cell dormancy secreting the same signals used to regulate quiescence of hematopoietic stem cells (HSCs) (Taichman et al., 2013; Yu-Lee et al., 2018; Ren et al., 2019; Yu-Lee et al., 2019). Indeed, Shiozawa *et al.* demonstrated in a mouse model of metastasis that human prostate cancer cells colonize the marrow osteoblastic niche by directly competing with HSCs (Shiozawa et al., 2011). Among many other molecules, osteoblasts produce the growth arrest specific 6 (GAS6) protein, which binds to the tyrosine kinase receptor Axl (Taichman et al., 2013). Interestingly, disseminated tumor cells frequently show high expression of the Axl receptor and become growth arrested in response to GAS6 (Taichman et al., 2013; Yumoto et al., 2016).

Just like in bone remodeling, osteoblasts and osteoclasts have opposite role also in tumor dormancy. While osteoblasts are primarily associated with dormancy induction and maintenance, osteoclasts have been reported in reactivation of dormant cells and generation of osteolytic metastases. Thus, tumor dormancy is a reversible state controlled by the extrinsic bone microenvironment. In this regard, the process of osteoclastic bone resorption leads to changes in the cellular composition and signaling within the bone marrow, which can cause the exit of cancer cells from a dormant state. Indeed, treatment of multiple myeloma-bearing mice with soluble RANKL to stimulate osteoclast formation and resorption resulted in a significant decrease in the number of dormant tumor cells in the bone marrow. The observation that myeloma cells in the spleen were not affected by the RANKL treatment confirmed that the effect on dormant cells was a non-cell-autonomous effect mediated by osteoclasts in the bone (Lawson et al., 2015). Accordingly, patients with recurrent myeloma show increased serum levels of C-terminal telopeptide (CTX), a biochemical marker of bone resorption (Lawson et al., 2015). Mechanistically, osteoclast-mediated bone resorption releases several growth factors, including TGF-β and periostin, which are tumor-promoting factors (Buijs et al., 2012). Therefore, osteoclasts are crucial to reactivation of tumor cells from dormancy in the process of bone metastasis.

## Targeting Osteoclasts to Limit Tumor Progression

The evidence that osteoclasts are key players in the formation of skeletal tumors provides the rationale for using antiresorptive drugs in the treatment of bone tumors and metastases. Hence, therapies commonly used to treat patients with bone remodeling disorders, for example, Paget’s disease of bone, have been translated in the clinical practice of bone cancers to mitigate the vicious cycle (Mackiewicz-Wysocka et al., 2012; Satcher and Zhang, 2021). As described before, one of the most utilized molecules belongs to the class of bisphosphonates, which target the bone matrix by binding to hydroxyapatite crystals (Zhang et al., 2007; Russell et al., 2008; Zielinska et al., 2021). Once this drug has been internalized by bone-resorbing osteoclasts, bisphosphonate inhibits their polarization and cytoskeleton rearrangement, thus compromising bone erosion and inducing their apoptosis (Wang L. et al., 2020). Currently, a frequently administrated bisphosphonate drug is zoledronic acid (ZA), which interferes with the mevalonic pathway, involved in the synthesis of steroids, such as cholesterol (Thurnher et al., 2012; Gobel et al., 2016). By inhibiting the farnesyl pyrophosphate synthase enzyme, ZA triggers the stop of posttranslational modifications of proteins and small GTPases, such as Rho, Ras, and Rac, thus inducing osteoclast apoptosis *via* destruction of the actin cytoskeleton structure (Gobel et al., 2016). In addition to jeopardizing osteoclast activity, ZA also directly interferes with the life span of cancer cells within the bone. Indeed, ZA activates the caspase-dependent apoptosis pathway to kill cancer cells, blocking Ras-dependent Erk 1/2 and Akt pathways, and then reduces the phosphorylation of Bcl-2 and Bad proteins to increase the apoptotic function (Tassone et al., 2003; Wang L. et al., 2020). To overcome the adverse effects of prolonged and massive use of bisphosphonates, lower concentrations of the drug are used in combination with adjuvants. For example, the simultaneous administration of ZA and the adjuvant anticancer drug ifosfamide—used for the treatment of human OS—showed decreased cancer-induced osteolytic lesions as well as efficient tumor growth arrest in a rat-transplantable model of osteosarcoma (Heymann et al., 2005).

In addition to bisphosphonates, other drugs are widely used to treat cancer-related osteoclastogenesis and to counteract metastasis formation. Denosumab is a fully human monoclonal antibody (IgG2) highly specific for RANKL and able to prevent its interaction with the receptor RANK, thereby blocking the osteoclastogenic process (Baron et al., 2011). Unlike other molecules interfering with RANK and RANKL binding (e.g., OPG-Fc), denosumab shows higher specificity to the substrate, a longer half-life, and no neutralizing antibodies have been identified (Baron et al., 2011). Interestingly, denosumab demonstrated superiority over ZA in preventing bone lesions in both breast and prostate cancers that metastasized to bone, by mimicking OPG action and minimizing pre-osteoclast and osteoclast survival and activity (Stopeck et al., 2010; Fizazi et al., 2011; Lipton and Goessl, 2011).

Additional molecules have been described as effective in limiting bone metastases by targeting osteoclast activity rather than osteoclasts themselves. Given the aforementioned role of TGF-β as a released factor promoting cancer proliferation, it is not surprising that TGF-β inhibitors represent another class of novel molecules utilized to prevent bone metastases, blocking the vicious cycle between cancer cells and the bone (Buijs et al., 2012; Hu et al., 2012; Wan et al., 2012). Neutralizing TGF-β antibodies have been developed to target individual ligands and all three TGF-β isomers (Baselga et al., 2008; Biswas et al., 2011). Athymic mice inoculated with MCF-7 breast cancer cells and then treated with neutralizing anti-TGF-β antibodies displayed total abrogation of cancer growth and metastasis (Arteaga et al., 1993). Notably, the use of these inhibitors does not affect the osteoblastic compartment, therefore preserving bone volume and architecture (Biswas et al., 2011). Likewise, inhibitors targeting integrin β3 have been developed for the treatment of breast cancer metastases to bone because of high expression of this molecule by breast cancer cells and its association with promoting skeletal tumor metastasis (Desgrosellier and Cheresh, 2010; Kovacheva et al., 2021). Of interest, by exploiting the specific overexpression of integrin β3 in the metastatic site, these molecules might be delivered in conjugation with nanoparticles that selectively target cancer cells (Ross et al., 2017). Recently, an emerging immunotherapy based on the use of nivolumab, an anti-PD-1 monoclonal antibody, has evidenced positive effect on tumor suppression and tumor-induced osteolysis (Wang K. et al., 2020). In fact, neoplastic cells produce high levels of PD-1 ligand, and monocytes/macrophages and pre-osteoclast present within bone tumor microenvironment express high levels of the PD-1 receptor. The binding of PD-1 ligand to its receptor leads to JNK activation and chemokine C-C motif ligand 2 (CCL2) secretion, thus promoting osteoclastogenesis. Strikingly, tumor-bearing mice treated with nivolumab showed total abolishment of bone osteolytic lesions, even though the tumor growth and progression were not totally neutralized (Wang K. et al., 2020). Patients with advanced-stage cancer experience intense pain owing to bone fractures or lesions as a consequence of bone metastases and accelerated osteolysis. To counteract these detrimental conditions, the use of STING (stimulator of interferon genes) agonists reduces bone cancer–induced pain and, equally important, through the induction of the STING/IFN-1 signaling, allows protection against bone destruction and tumor growth (Wang et al., 2021).

A common challenge for all the aforementioned therapies is to face potential toxicity caused by the pleiotropic roles of the targeted pathways. A new therapeutic approach could benefit from the positively charged bone matrix of the acidic environment. For example, conjugation of antibodies or inhibitors with bisphosphonates, which are negatively charged, can significantly enrich these molecules in the bone microenvironment, thereby reducing side effects on other organs (Cole et al., 2016; Farrell et al., 2018; Tian et al., 2021).

## Conclusion

A widely accepted view in biology implies osteoclasts as highly specialized cells quite exclusively implicated in bone resorption during the remodeling. However, although never described as the neoplastic cell, several studies agree that the osteoclast influences the development, progression, and aggressiveness of bone tumors, both primary and metastatic. Therefore, better understanding of the molecular mechanisms governing the osteoclast function may result in the development of novel diagnostic and therapeutic approaches. Here, we underline that the skeleton should not be overlooked in patients with primary tumors in other sites (e.g., breast and pancreas), even when the mass has been completely eradicated through pharmacological or surgical approaches. Indeed, the osteoclast-mediated bone resorption activity might promote either the growth of metastatic cells within the marrow or the reactivation from tumor dormancy. Serum markers of bone resorption should be routinely tested in periodic follow-ups for the assessment of osteoclast activity in patients with bone tumors or metastases. In conclusion, we highlight that the osteoclast could be considered as a pro-cancer cell due to its ability to degrade bone matrix and release tumorigenic factors, thus creating a pro-tumoral microenvironment.
